# Predicting Sexual Behaviors Among Homeless Young Adults: Ecological Momentary Assessment Study

**DOI:** 10.2196/publichealth.9020

**Published:** 2018-04-10

**Authors:** Diane Santa Maria, Nikhil Padhye, Yijiong Yang, Kathryn Gallardo, Michael Businelle

**Affiliations:** ^1^ School of Nursing University of Texas Health Science Center at Houston Houston, TX United States; ^2^ School of Public Health University of Texas Health Science Center at Houston Houston, TX United States; ^3^ Department of Family and Preventive Medicine The University of Oklahoma Health Sciences Center Oklahoma City, OK United States

**Keywords:** homeless youth, sexual behaviors, ecological momentary assessment

## Abstract

**Background:**

Homeless youth continue to be disproportionately affected by HIV compared with their housed peers, with prevalence rates as high as 13%. Yet, HIV prevention in this high-risk population has been only marginally effective.

**Objective:**

The aim of this study was to use ecological momentary assessments to examine real-time factors to determine the predictors of sexual activity among homeless youth.

**Methods:**

Youth experiencing homelessness aged between 18 and 24 years were recruited from a drop-in center in Houston, Texas, between August 2015 and May 2016. All the participants received a study-issued mobile phone that prompted brief ecological momentary assessments (EMAs) 5 times a day for 21 days. EMA items assessed near real-time sexual behaviors, cognitions, stress, affect, environmental factors, and environmental circumstances.

**Results:**

Participants (N=66) were predominantly male (41/66, 64%) and black (43/66, 66%) with a median age of 20 years. The mean number of EMAs completed by each participant was 45 out of 105 possible observations. During the study, 70% (46/66) of participants were sexually active and reported condomless sex in 102 of the 137 cases of sexual intercourse (74.5%). In total, 82% (38/46) of the youth who reported having sex during the 3 weeks of data collection also reported engaging in high-risk sexual activities, including having condomless sex (24/46, 53%), having multiple sexual partners on the same day (12/46, 26%), trading sex (7/46, 16%), and sharing needles while injecting drugs (1/46, 3%). Of those, 71% (27/38) were engaged in multiple sexual risk behaviors. The predictive model was based on observations from 66 subjects who reported 137 cases of sexual intercourse over 811 days; sexual orientation, race, mental health, drug use, and sexual urge were included as predictors in the parsimonious generalized linear mixed model selected on the basis of the Akaike information criterion. The estimated odds ratios (ORs) were notable for same-day drug use (OR 8.80, 95% CI 4.48-17.31; *P*<.001) and sexual urge (OR 4.23, 95% CI 1.60-11.28; *P*=.004). The performance of the risk estimator was satisfactory, as indicated by the value of 0.834 for the area under the receiver operating characteristic curve.

**Conclusions:**

Real-time EMA data can be used to predict sexual intercourse among a sample of high-risk, predominately unsheltered homeless youth. Sexual urge and drug use accounts for increased odds of engaging in sexual activity on any given day. Interventions targeting sexual urge and drug use may help predict sexual activity among a population at high risk of HIV.

## Introduction

### Background

On any given night in the United States, 1.7 to 2.5 million youth under the age of 25 years are homeless [1-3]. Homeless youth are 6 to 12 times more likely to become infected with HIV than housed youth [4], with prevalence rates as high as 13% [5]. Homeless youth are also at greater risk for sexually transmitted infections (STIs) than their housed peers, with 23% of homeless youth reporting having an STI [6]. These high prevalence rates can be partially attributed to the high prevalence of sexual risk behaviors. Homeless youth, become sexually active at an earlier age, are more likely to have multiple sex partners; to trade sex for food, shelter, money, or substances [7-9]; and to use substances before sex. However, they are less likely to use a condom than stably housed youth [10,11]. Youth are also more likely to engage in these high-risk sexual behaviors when they use substances [12]; substance use is high in the homeless youth population [13].

### Challenges of Sexual Risk Behavior Research Among Youth Experiencing Homelessness

For decades, prevention research has been challenged by the unsubstantiated belief that homeless youth are beyond help [14]. Prevention interventions specifically targeting homeless youth, though rare, have achieved marginal and temporary improvements in sexual health outcomes (eg, decreased frequency of condomless sex in females) and have been limited to substance-using samples [4,15-18]. However, even modest intervention effects in a marginalized understudied, high-risk population moves the science forward [19]. The disappointing speed at which advancements in prevention research for homeless youth have evolved may be due to the challenges of conducting research among homeless youth [16]. Likewise, our lack of understanding of how real-time factors such as sexual urge, substance use, and stress influence sexual risk behaviors may further stall scientific advancements in this area [20]. The unique experience of homelessness creates significant challenges that need to be addressed such as low concern for STIs, high-risk sexual behaviors [21,22], and the high levels of stress associated with meeting basic needs for food and shelter [11]. Although gender identity, age, race and ethnicity, sexual orientation, educational attainment, and adverse childhood experiences are all linked to sexual risks among homeless youth [23,24], the prevalence of risk behaviors is also elevated by the circumstances experienced before and subsequent to becoming homeless such as sexual abuse and victimization [25-27]. To this end, understanding how the unique extenuating circumstances that precede and extend into homelessness affect one’s thoughts, feelings, and environment, and influence sexual behavior decision making in real time is needed.

### Using Ecological Momentary Assessments in Prevention Research Among Youth Experiencing Homelessness

Recall data have a higher potential for bias, neglect intraindividual variability, and do not capture risk and predictive factors as they occur in real-world settings. Ecological momentary assessment (EMA) allows for the examination of within-person variance in risk exposures (ie, where, when, and with whom sexual risk is likely to occur throughout a day) by capturing repeated measures to assess changes in behaviors, cognitions, environmental factors, and symptoms [28,29]. EMA has been used to assess sexual behaviors [30] and drinking in young people [31]. To date, no studies have used EMA to assess whether real-time factors can be used to predict sexual behaviors among homeless youth. EMA may be an effective strategy to gain a better understanding of how real-time thoughts, feelings, and environmental factors affect sexual risk behaviors. To further the science of HIV prevention in homeless youth, strategies must consider the transient nature of being homeless and the varying daily circumstances that influence real-time sexual urge, substance use, stress, and risky decision making. Risk behaviors that are associated with HIV are related to the daily experiences of vulnerability and stress associated with homelessness [27]. The EMA approach is currently the gold standard and most accurate way to measure real-time factors in natural settings [28,32] with high compliance rates (78%) found among youth across 42 studies [33]. High EMA completion rates have been found in other studies on substance using Latino youth (80%) [34], youth in recovery (87%) [35], and youth smokers (88%) [36]. EMA data that are collected at or near the moment when behaviors occur can reduce memory bias and other biases that are associated with retrospective recall measures.

### Theoretical Framework

HIV prevention interventions for homeless youth may differ significantly from those for other youth owing to the extenuating circumstances of homelessness. This study was guided by the Risk Amplification Model, which posits that sexual risk behaviors of homeless youth are elevated by the circumstances experienced before and subsequent to homelessness [25-27]. Therefore, there’s a need to understand the role of stress, urge, substance use, and current homeless issues’ impact on HIV risk [37,38].

### Purpose

The purpose of this study was to use EMA data to examine real-time factors such as stress, urge, and substance use to determine the predictors of sexual activity among homeless youth. Specifically, the objectives of the study were to determine whether EMA data from among homeless youth can be used to predict sexual risk behaviors.

The primary research question that guided this study was: what are the predictors of sexual intercourse among homeless youth? We hypothesize that real-time factors will predict sexual intercourse. We report the findings of this study using the adapted Strengthening the Reporting of Observational Studies in Epidemiology Checklist for Reporting Ecological Momentary Assessment Studies, which includes the reporting of sampling strategies, measures, schedule, technology used, administration, participant prompting strategy, response rate, and compliance rate [39].

## Methods

**Participant Recruitment**

**Figure 1:**
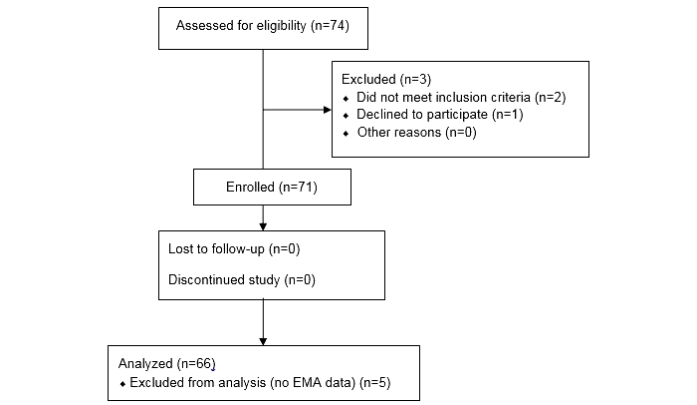
CONSORT (Consolidated Standards of Reporting Trials) E-HEALTH Flow Diagram. EMA: ecological momentary assessment.

Homeless youth aged between 18 and 24 years were all recruited through study information sessions at shelters and the largest homeless youth drop-in center in Houston, Texas. Flyers were posted at shelters and drop-in centers to advertise for the information sessions. Participants who responded to study advertisements and approached the study team at the drop-in center were provided with the details of the study, and their interest in participating was assessed (see Figure 1). Interested individuals were briefly screened for study eligibility. Participants were included if they were homeless, 18 to 24 years old, English speaking, and able to participate for the duration of the study period (ie, not planning to move out of the county during the study). Homelessness was defined as sleeping on the streets, in a place not meant for human habitation, in a shelter, in a hotel or motel, or with someone with whom they could not stay for more than 30 days (ie, couch surfing). Individuals were excluded if they had very low literacy, owing to the need to be able to read and understand EMAs unassisted throughout the study [40]. Low literacy was defined as a score of less than 4 on the Rapid Estimate of Adult Literacy in Medicine-Short Form [40,41], an interviewer-administered checklist in which individuals are asked to read and pronounce 9 common medical terms. Individuals who pronounce ≥4 words correctly are considered to be reading at >6th grade reading level.

**Study Procedures**

Details of the study were discussed with eligible youth, who then provided written informed consent, witnessed by the study staff, and received a study summary and a copy of the informed consent document. After the initial eligibility screening, participants completed an audio-assisted baseline survey on an Apple iPad using Qualtrics. The baseline survey took approximately 30 min to complete and assessed demographics such as gender identity, age, race/ethnicity, sexual orientation, educational attainment, and adverse childhood experiences. Participants were then provided with a study-issued mobile phone, with instructions for using the phone and accessing and completing the EMAs over the 21-day study duration. Each participant was asked to indicate his/her normal waking hours to ensure that EMA prompts would not wake the participant. Snacks and beverages were provided and breaks were encouraged as needed. Participants received a US $20 gift card for completing the baseline survey and were provided with bus tickets or METRO pass when needed to cover the cost of local transportation on baseline and exit study visits.

Study staff contacted participants on their study phone to schedule the final study visit 21 days after the initial study visit. Participants were asked to meet at a local drop-in center, a shelter, or a local library to complete an exit survey, return the study mobile phone, and receive grocery store gift cards. Upon returning the study smartphone at the final visit, participants received up to $95 in gift cards. The amount of compensation depended upon the percentage of random and daily EMAs completed. Specifically, participants who completed 49.5% (52/105) to 75.2% (79/105) of EMAs received a $50 gift card, those who completed 76.2% (80/105) to 88.6% (93/105) of EMAs received a $75 gift card, and those who completed 89.5% (94/105) or more of EMAs received a $95 gift card. Those who completed <50% of assessments received a $20 gift card for returning the phone. This incentive structure was explained to all participants during the informed consent process. Participants were able to access their current compensation level throughout the study period through the study-issued phone interface.

### Baseline Measures

We assessed age, race/ethnicity, sexual orientation, and mental illness at baseline. To assess race/ethnicity, youth were asked if they identified as black, white, Asian, Hispanic, American Indian, multiracial, or something else. We created a category called “Other” that included those who identified as American Indian or Alaska Native, multiracial, or something else. Sexual orientation was measured by asking youth if they identified as heterosexual, gay, lesbian, bisexual, or something else. Mental illness was assessed by asking youth if they had ever been diagnosed with attention deficit-hyperactivity disorder (ADD/ADHD), depression, bipolar disorder, psychosis, schizophrenia, oppositional defiant disorder, conduct disorder, or posttraumatic stress disorder (PTSD).

**Ecological Momentary Assessment Measures Schedule**

EMAs were used to collect data in near real time using study-provided mobile phones. The EMA methodology used is similar to that developed by Shiffman, Stone, and colleagues [29,42,43] and has been used by our study team in multiple studies [44-46]. EMAs were prompted 5 times a day. Time-based sampling (daily diary) and random sampling EMAs were completed. The phone audibly and visually cued each assessment for 30 seconds. If the participant did not respond after 3 prompts, the assessment was recorded as missed. Daily diary assessments were prompted once every day 30 min after the participant’s indicated normal waking time. Questions referred to the previous 24 hours and queried about risk behaviors. The daily EMAs assessed items such as sheltering, and engaging in sexual activity, substance use, and alcohol consumption. Shelter day referred to youth who had spent the night in a shelter versus on the street or staying with someone temporarily. Questions such as “Did you have sex yesterday?,” “Did you trade sex yesterday?,” “Did you drink any alcoholic beverages yesterday?,” and “Did you view pornography yesterday?” were used to assess behaviors. If the participant answered “yes” to the sexual activity item, the participant was prompted to indicate the type of sexual activity (eg, oral, vaginal, or anal), whether a condom was used, and the number, and gender of the sexual partner or partners. The 4-item Perceived Stress Scale was used to measure stress. Scores were summed, with higher scores indicating more stress. Daily diary assessments took less than 5 min to complete. For the main outcome of sexual activity, each incident of sexual activity as indicated on the daily EMA, was considered a positive case.

Random assessments were scheduled to occur randomly in 4 epochs during each participant’s normal waking hours. Random EMAs took approximately 2 min to complete. In the random EMAs, participants rated their current affect by indicating the extent to which they felt irritable, happy, content, frustrated or angry, sad, worried, miserable, restless, stressed, hostile, and calm. Behaviors were assessed by asking about substance use and alcohol use. Sexual urge was measured by asking youth if they were feeling a strong urge to have sex, use drugs or alcohol, or steal.

### Technology and Hardware

The Samsung Galaxy Light mobile phone with the Android 4.2 operating system was used to send EMAs. Participants could call and receive calls from research staff through the smartphone free of charge.

### Data Analysis

R, package lme4, and package pROC (R project) were used in the analyses [47-49]. Generalized linear mixed models (GLMMs) were used to model the high-frequency longitudinal EMA data with a logistic link used for adaptation to the binary outcome of sexual intercourse. A random intercept was added to account for correlated observations within subject. The identified predictors of the dependent variables included EMA data that preceded the case behavior and assessed real-time sexual urge, substance use, and stress up to 24 hours before the occurrence of sexual intercourse. To maximize the use of available data, we built models in 3 steps. Missing data were not estimated but were ignored to avoid the introduction of bias due to the estimation method. Time-invariant predictors (ie, demographic data) were included in the first step. The Akaike information criterion (AIC) [50] was used to systematically eliminate variables that were not predictive with backward selection. Daily event data were introduced in the second step, and predictors were similarly eliminated using AIC for model selection. Random EMA data were summarized by day to match the collection frequency of the outcome before being introduced in the third step of modeling. Model performance was assessed with a receiver operating characteristic (ROC) curve and with cross-validated sensitivity and specificity. Cross-validation was accomplished over the course of 100 runs with 80/20 random splits of training and test sets [51]. For measuring sensitivity and specificity, the model estimates of the probabilities of sexual intercourse events were converted to binary predictions of sex events (yes or no) with the choice of a decision threshold, (eg, sex event predicted when the probability of sexual intercourse exceeded 0.3). The decision threshold was chosen to provide a reasonable balance between true positive and false positive rate, which was measured with the likelihood ratio.

## Results

### Sample Characteristics

The mean age of the sample was 21.2 years, with 55% (36/66) aged between 21 and 24 years. The majority of participants were male (41/66, 62%), black (43/66, 65%) or other race (16/66, 24%), and a minority (14/66, 21%) identified as lesbian, gay, bisexual, transgender, or questioning (Table 1). The median age at the onset of homelessness was 16.9 years. Only 13.4% (108/806) of days were shelter days, with most days being on the streets (293/806 days) or staying with someone temporarily (405/806 days). At baseline, 20% (13/66) of participants indicated a history of having an STI diagnosis. Among the total sample, 73% (48/66) reported at least 1 mental health diagnosis (ADHD, bipolar disorder, depression, oppositional defiant disorder, conduct disorder, psychosis, schizophrenia, or PTSD). Of these, 11% (7/66) had a diagnosis of psychosis, 21% (14/66) reported PTSD diagnosis, and 46% (30/66) reported a diagnosis of bipolar disorder, manic depression, or depression. More than half of the participants reported having a diagnosis of ADD/ADHD.

### Response and Compliance Findings

We received EMA data from 66 of 71 recruited participants, indicating a 93% participation rate (Table 2). Table 2 provides the number and percentage of surveys that endorsed the variable, number of EMAs included in each count, and the number of participants who provided EMA data by variable. The mean number of EMAs provided by each participant was 45 observations—that is, 13.0 out of 21 (61.9%) of daily EMAs and 33.6 out of 84 (40%) of random EMAs.

The average daily EMA compliance rate of 61.9% conservatively assumes that all participants received all of the EMA over the 21 days for a total of 105 (Table 3). However, due to sheltering instability among the sample, participants reported frequent loss of phone battery charge due to having no available electrical outputs. This likely decreased the actual number of EMAs received. For the random data, each participant had the potential to receive 4 random surveys per day for 21 days. Conservatively, the average number of random EMAs per participant was 33.6 out of 84, indicating a random EMA average compliance rate of 40.0%. The compliance rate of daily EMAs across all was higher than the compliance rate of random EMAs. Participants who identified as other race, female, and older than 20 years of age completed more random EMAs than their counterparts. Random EMA completion rates were similar across sexual orientations.

**Table 1:** Participant demographics and sexual behaviors. LGBT: lesbian, gay, bisexual, and transgender; ADD/ADHD: attention deficit-hyperactivity disorder; ODD: oppositional defiance disorder; CD: conduct disorder; PTSD: posttraumatic stress disorder.

Demographics		Participants	Condomless sex^a^	Multiple sexual partners^a^	Trade sex^a^	Substance use^a^	Alcohol use^a^
Participants, n		66	35	17	11	40	21
**Age, years, n (%)**							
	≤20	30 (46)	22 (63)	8 (47)	7 (64)	20 (50)	7 (33)
	>20	36 (55)	13 (37)	9 (53)	4 (36)	20 (50)	14 (67)
**Gender identity, n (%)**							
	Male	41 (62)	19 (54)	11 (65)	4 (36)	25 (63)	11 (52)
	Female	24 (36)	15 (43)	6 (35)	7 (64)	14 (35)	9 (43)
	Other^b^	1 (2)	1 (3)	0 (0)	0 (0)	1 (3)	1 (5)
**Race, n (%)**							
	Black	43 (65)	23 (66)	13 (77)	8 (73)	25 (63)	14 (67)
	White	7 (11)	2 (6)	1 (6)	0 (0)	4 (10)	2 (10)
	Other^c^	16 (24)	10 (29)	3 (18)	3 (27)	11 (28)	5 (24)
**Ethnicity, n (%)**							
	Hispanic	8 (12)	4 (11)	2 (12)	2 (18)	4 (10)	3 (14)
**Sexual orientation, n (%)**							
	Heterosexual	52 (79)	24 (69)	10 (59)	3 (27)	28 (70)	11 (52)
	LGBT	14 (21)	11 (31)	7 (41)	8 (73)	12 (30)	10 (48)
Foster History, n (%)		22 (34)	39 (38)	14 (45)	10 (59)	85 (41)	24 (55)
**Mental health diagnosis, n (%)**							
	ADD or ADHD	37 (56)	23 (66)	12 (71)	7 (64)	26 (65)	14 (67)
	Bipolar or manic	30 (46)	18 (51)	9 (53)	7 (64)	19 (48)	11 (52)
	Depression	30 (46)	18 (51)	7 (20)	6 (55)	20 (50)	12 (57)
	ODD or CD	13 (20)	9 (26)	7 (20)	3 (27)	10 (25)	6 (29)
	Psychosis	7 (11)	3 (9)	2 (6)	2 (18)	4 (10)	2 (10)
	PTSD	14 (21)	9 (26)	5 (14)	3 (27)	8 (20)	4 (19)

^a^Number of participants who engaged in the specified behavior at least once.

^b^Other: transgender, nonbinary gender.

^c^Other: American Indian or Alaska Native; Multiracial; Something else.

**Table 2:** Ecological momentary assessment (EMA) and participant count and frequency by variable.

Variable	EMA surveys endorsing^a^, n (%)	Participants, n (%)^b^
Sexual activity	137 (16.9)	46 (70)
Multiple sexual partner	31 (22.1)	17 (26)
Condomless sex	102 (74.5)	35 (53)
Trade sex	17 (12.4)	11 (17)
Substance use	210 (26.1)	40 (61)
Sexual urge	124 (15.5)	44 (67)
Alcohol use	44 (6.7)	21 (32)
High stress day	235 (29.0)	47 (71)
Pornography use	152 (17.7)	43 (65)
Shelter day	108 (13.4)	4 (6)

^a^Number and percentage of the total number of surveys that endorsed this behavior.

^b^Number of participants of the total 66 participants who engaged in the behavior at least once.

**Table 3:** Ecological momentary assessment (EMA) compliance rates by demographics. LGBT: lesbian, gay, bisexual, and transgender.

Variable		Total EMA (n=105), mean (SD)	Daily EMA (n=21), mean (SD)	Random EMA (n=84), mean (SD)
Whole sample		46.7 (28.52)	13.03 (6.32)	33.6 (22.8)
**Race**				
	Black	45.47 (28.72)	12.91 (6.51)	32.56 (22.8)
	White	46.71 (29.91)	12.86 (7.08)	33.86 (23.46)
	Other	50.6 (28.95)	13.44 (5.89)	23.88 (23.88)
**Gender**				
	Male	42.08 (24.83)	12.27 (6.16)	29.55 (19.45)
	Female	52.88 (32.60)	14 (6.51)	38.88 (26.32)
	Other	89 (-)	21 (-)	68 (-)
**Age, years**				
	≤20	43.83 (26.81)	12.43 (5.85)	31.03 (21.61)
	>20	49.17 (30.00)	13.53 (6.74)	35.64 (23.77)
**Sexual orientation**				
	Heterosexual	46.82 (28.36)	12.96 (6.25)	33.65 (22.66)
	LGBT	46.64 (30.17)	13.29 (6.84)	33.36 (24.05)

**Table 4:** Generalized linear mixed models (GLMM) coefficients and odds ratios for predictors of sexual intercourse. OR: odds ratio; LGBT: lesbian, gay, bisexual, and transgender; PTSD: posttraumatic stress disorder.

Variable	Coefficient B	SE	OR	Z	*P* value	95% CI of OR
Fixed effects						
Intercept	−2.846	0.576	0.06	−4.944	<.001	0.019-0.180
Sexual orientation (LGBT)^a^	0.8703	0.4061	2.388	2.143	.03	1.077-5.290
Race (white)^a^	−0.7501	0.6724	0.472	−1.116	.27	0.127-1.763
Race (other)^a^	0.9205	0.4207	2.511	2.188	.03	1.101-5.733
Psychosis^a^	1.4716	0.6195	4.356	2.376	.02	1.293-14.690
PTSD^a^	−1.6613	0.4681	0.190	−3.549	<.001	0.076-0.475
Drug use	2.1748	0.3445	8.800	6.313	<.001	4.476-17.309
Sexual urge	1.4431	0.4999	4.234	2.887	.004	1.589-11.280

^a^Reference group is black, heterosexual youth without mental illness.

#### Ecological Momentary Assessment

In total, 66 participants completed 860 daily EMAs (Table 1). We analyzed the daily EMAs descriptively and found that work and school days among the sample were low (6% each). Of the 66 participants, 38 (58%) reported engaging in high-risk sexual behaviors during the data collection period, including having condomless sex, having multiple sexual partners in the same day, trading sex, or sharing needles to inject drugs. Of those 38 youth, 26 (71%, 26/38) had engaged in more than one of the risk behaviors during the study. Substance use rates were also high, 62% (40/66) and 32% (21/66) reported using drugs and alcohol, respectively.

Although 70% (51/66) of the participants were sexually active during the study, 53% (35/66) reported condomless sex accounting for 102 incidences or 75% of sexual intercourse incidences. Additionally, 26% (17/66) had sex with more than one person in a day, 16% (11/66) engaged in trade sex, and 18% (12/66) used pornography during the 3-week EMA period. Pornography use appeared to be a cluster behavior with other risk behaviors occurring on the same day as 40% of condomless sex days, 68% of multiple sexual partner days, 65% of trade sex days, and 46% of substance use days. Sexual urge was experienced by 67% of the participants and was reported on 124 or 15% of daily EMA study days. Higher rates of sexual intercourse were reported on high sexual urge days and drug use days.

#### Risk Estimator for Sexual Activity Days

The final predictive model for sexual intercourse included both between- and within-subject variables: race, sexual orientation, mental illness, drug use, and sexual urge in the parsimonious GLMM selected on the basis of the AIC (Table 4). The risk estimator was based on observations from 66 participants over 860 days and included 137 days of sexual intercourse. Of note, not all predictors had significant *P* values, factors needed to be included to minimize the AIC, indicating that they were not ignorable in predicting substance use. The estimated odds ratios (ORs) for the within-subject predictors were notable for 2 states; drug use (OR 8.80, *P*<.001) and sexual urge (OR 4.23, *P*=.004). The odds of having sex increase 8.8 times on days when youth use drugs after adjusting for sexual urge and other predictors. The odds of having sex increase 4.2 times per unit increase in sexual urge, after adjusting for drug use and other predictors.

The performance of the risk estimator was very good, as indicated by the value of 0.834 for the area under the ROC curve [52]. The cross-validation run over 100 times with randomized 80/20 splits of the data into training and test sets resulted in a mean sensitivity of 0.17 and specificity of 0.96 with a likelihood ratio of 4.45 for a decision threshold of *P*=.48 (ie, sex predicted if predicted probability exceeds 0.5). For a decision threshold of *P*=.20 (ie, sex predicted if predicted probability exceeds 0.20), the sensitivity and specificity were 0.637 and 0.832, respectively with a likelihood ratio of 3.80.

Finally, we compared the model using only traits as predictors versus the addition of drug use and sexual urge. The area under the ROC curve dropped from 0.834 to 0.683, indicating that the model improves with the state variables as values below 0.7 are often considered unsatisfactory. Additionally, using a decision threshold of *P*=.20 to predict sex events, the cross-validated sensitivity dropped to 50.1%, the specificity dropped to 73.3%, and the likelihood ratio halved from 3.8 to 1.9.

## Discussion

The findings presented here represent a predominantly unsheltered and unstably housed sample of young adults with high-risk sexual behaviors and high rates of substance use. Sexual intercourse was predicted by both between- and within-subject variables, including real-time drug use and sexual urge. Subgroups of homeless youth emerged as higher risk for sexual intercourse. The odds of sexual intercourse were highest among nonwhite, other race youth, those that identify as lesbian, gay, bisexual, and transgender (LGBT), and those who have ever been diagnosed with PTSD. Therefore, special attention is needed to address HIV risk reduction within minority youth and those with mental illness.

Our findings suggest that the majority of the homeless youth in this study were sexually active and they primarily engage in condomless sex, placing them at heightened risk for HIV, STIs, and pregnancy. While previous studies have demonstrated the high rates of sexual risk behaviors among homeless youth [8,11,53], the majority of those studies used a cross-sectional study design and assessed the occurrence of sexual risk behaviors either by recall of behaviors within the past 1 to 3 months or by assessing condom use at last sex. These studies reported rates of condomless sex that ranged from 40% to 70% [54]. By using EMA in a high frequency longitudinal study design, we assessed behaviors in near real-time by asking participants to record sexual risk behaviors that occurred within the past 24 hours. This methodology has been shown to reduce recall biases that are commonly associated with cross-sectional retrospective recall measures. By asking youth to report condomless sex per instance of sexual intercourse in near real-time, we found that rates of condom use were much lower than those reported in studies using recall measures or assessing condom use during the last sexual intercourse.

Although characteristics of homeless youth suggest higher risk subgroups, one’s real-time urges and drug use also influence the odds of engaging in sexual intercourse. Youth experiencing homelessness are at higher risk of engaging in sexual intercourse on the days they use substances. In alignment with previous literature [11,13], we found that homeless youth engage in high rates of substance use. Approximately 60% of participants reported using substances and nearly one-third of participants reported using alcohol during the study period. More importantly, we found that homeless youth were more likely to engage in sexual intercourse on the days they reported using substances. Previous studies demonstrated that the use of drugs and alcohol can impair homeless youth’s sexual health decision-making capabilities, thus increasing their risk for engaging in sexual risk behaviors and acquiring HIV [12,21,55]. Additionally, the Risk Amplification Model suggests that homeless youth form high-risk social networks. Therefore, when youth reported unstable housing days, these relationships may represent high-risk social networks that may contribute to the risk for substance use.

We found that youth are at higher odds of engaging in sexual intercourse on days when they experience sexual urges. Kennedy and colleagues found that experiencing feelings of intense sexual arousal influenced homeless youth’s decision to engage in unprotected sex [22]. Thus, our results align with the research that experiencing sexual urges is a risk factor for subsequently engaging in sexual intercourse and that sexual urge can be detected in real time using EMA.

The results of our study also indicated that pornography use was common among homeless youth, with approximately 65% of participants reported viewing pornography at least once during the study period. Interestingly, viewing pornography appeared to be clustered with other risk behaviors, including having condomless sex, trading sex, and having multiple sexual partners on the same day. Our findings support previous research examining the impact on sexual risk behaviors of pornography use among youth and young adults [56,57]. For example, Braun-Courville and Rojas found that youth who viewed pornography were more likely to engage in sexual risk behaviors [57]. This represents an understudied area among homeless youth that warrants more attention.

### Significance of the Findings

This is the first study to use EMA data to predict the likelihood of engaging in sexual intercourse in near real time among a high-risk, hard-to-reach, homeless youth population. Using EMA, we found that 26% (17/66) of youth experiencing homelessness had multiple sexual partners in a day, and about 53% (35/66) engaged in condomless sex which likely contributes to the high rate of HIV and STIs among homeless youth. Additionally, the EMA and statistical analysis methods used here are potentially applicable to other hard-to-reach populations and can be used to predict other risk behaviors that occur with frequency and are potentially affected by real-time cognitions and behaviors.

### Implications for Research and Prevention Interventions

This study demonstrates that it is possible to predict days when youth are at higher risk for engaging in sexual intercourse. To this end, it may be possible to develop just-in-time interventions that can disrupt the progression from drug use and sexual urge to engaging in sexual risk behaviors by addressing drug use and identifying skills to manage sexual urge in ways that reduce sexual risks. If we can predict days when youth are at higher risk of having sexual intercourse, we can design and test safer sex promoting motivational messaging that can be delivered at the time of heightened risk and have the potential to enhance safer sexual behavior decision making. For example, since these data revealed that 75% of sexual acts were condomless, messages encouraging condom use could be sent to participants on the days when youth report sexual urge or drug use.

### Limitations

The findings represented here may not reflect other possible real-time predictors that were not measured. Although we constructed a comprehensive EMA survey based on extensive formative research [58-60] that included variables indicated in the literature to affect sexual behaviors, other variables may also influence real-time sexual risk. That said, using EMA to predict risk behaviors is a relatively novel scientific method that does not have defined guidelines for best practices for measurement, implementation, or analysis, particularly among vulnerable populations such as homeless youth. In so far as the EMA approach is an emerging science, the measures used to assess real-time factors have not yet been psychometrically validated. The temporality of the data is another limitation. EMA improves on the ability to assess subsequent behaviors from real-time measures. However, participants reported their sex behaviors from the preceding day. Drug use and sexual urge EMA variables were calculated for the preceding day to align with the sexual behavior variable. Therefore, we cannot unequivocally conclude, on the basis of these data, that drug use and urge preceded sexual intercourse on a given day. Finally, though we used statistical methods to cross-validate the predictive model, a subsequent study in a new population of homeless youth to test the model would provide further validation.

### Conclusions

High frequency longitudinal EMA data that assess real-time factors can be used to predict sexual intercourse. This kind of data and analyses can inform the design of just-in-time adaptive interventions that could be delivered using mobile phones to deliver health promoting and motivational messaging at the time of heightened risk.

## Abbreviations

ADHD: attention deficit-hyperactivity disorder
AIC: Akaike information criterion
EMA: ecological momentary assessment
GLMM: generalized linear mixed models
LGBT: lesbian, gay, bisexual, and transgender
OR: odds ratio
PTSD: posttraumatic stress disorder
ROC: receiver operating characteristic curve
STI: sexually transmitted infection
