# Correction: Predicting Sexual Behaviors Among Homeless Young Adults: Ecological Momentary Assessment Study

**DOI:** 10.2196/10806

**Published:** 2018-05-07

**Authors:** Diane Santa Maria, Nikhil Padhye, Yijiong Yang, Kathryn Gallardo, Michael Businelle

**Affiliations:** ^1^ School of Nursing University of Texas Health Science Center at Houston Houston, TX United States; ^2^ School of Public Health University of Texas Health Science Center at Houston Houston, TX United States; ^3^ Department of Family and Preventive Medicine The University of Oklahoma Health Sciences Center Oklahoma City, OK United States

The authors of the paper “Predicting Sexual Behaviors Among Homeless Young Adults: Ecological Momentary Assessment Study” (JMIR Public Health Surveill 2018;4(2):e39) made an error in the reporting of odds ratios (ORs) and the labeling of the columns in Table 4. While it does not change the interpretation of the data or outcomes, the values need to be corrected.

In the Abstract, the OR values for same-day drug use and sexual urge in the sentence “The estimated odds ratios (ORs) were notable for same-day drug use (OR 2.17, 95% CI 4.48-17.31; *P*<.001) and sexual urge (OR 1.44, 95% CI 1.60-11.28; *P*=.004)” have been changed to 8.80 and 4.23, respectively.

In the “Risk Estimator for Sexual Activity Days” subsection of the Results, the OR values for same-day drug use and sexual urge have again been changed to 8.80 and 4.23, respectively. The subsequent text which previously read “The odds of having sex on a given day is more than double for every unit increase in drug use. The odds of having sex rise by 44% for every unit increase in sexual urge” has been changed to the following:

The odds of having sex increase 8.8 times on days when youth use drugs after adjusting for sexual urge and other predictors. The odds of having sex increase 4.2 times per unit increase in sexual urge, after adjusting for drug use and other predictors.

In Table 4, column 2 was mistakenly labeled “OR” and is now “Coefficient B”. Column 4 was mistakenly labeled “Exp(B)” and is now “OR”. The data values for these columns have not been changed. The correct header for the table appears below.

The corrected article will appear in the online version of the paper on the JMIR website on May 7, 2018, together with the publication of this correction notice. Because this was made after submission to PubMed, Pubmed Central, and other full-text repositories, the corrected article also has been re-submitted to those repositories.

**Table 4:** 

Variable	Coefficient B	SE	OR	Z	*P* value	95% CI of OR


